# Acute Gastric Volvulus: A Rare Complication of Hiatal Hernia

**DOI:** 10.7759/cureus.66102

**Published:** 2024-08-03

**Authors:** Sagar Nagpal, Hezborn M Magacha, Puneet Goenka, Venkata Vedantam

**Affiliations:** 1 Department of Internal Medicine, East Tennessee State University Quillen College of Medicine, Johnson City, USA; 2 Division of Gastroenterology and Hepatology, East Tennessee State University Quillen College of Medicine, Johnson City, USA

**Keywords:** diagnostic imaging, gastric perforation, surgical emergency, hiatal hernia, gastric volvulus

## Abstract

Gastric volvulus, characterized by stomach rotation, is a rare condition arising from congenital or acquired factors. Predominantly affecting pediatric and elderly populations, it necessitates a high index of suspicion for timely diagnosis. Delayed recognition may precipitate severe complications such as ischemia, strangulation, and septic shock, often culminating in fatal outcomes. We present a case of a 71-year-old male initially admitted for suspected gastroenteritis, subsequently developing acute gastric volvulus during hospitalization, necessitating immediate surgical intervention. This case contributes to the scant literature on gastric volvulus in the elderly demographic.

## Introduction

Gastric volvulus, a rare yet potentially life-threatening condition, entails the rotation of the stomach along its transverse or longitudinal axis by at least 180 degrees [1]. This rotation manifests in two forms: organoaxial and mesenteroaxial. Organoaxial rotation happens along the axis of the stomach, while mesenteroaxial rotation occurs perpendicular to this axis. Organoaxial rotation is more common, occurring in about 59% of cases, while mesenteroaxial rotation happens in about 29% of cases [2].

Volvulus can stem from primary or secondary causes. Primary causes involve laxity and disruption of the stomach's ligamentous attachments [3], while secondary volvulus arises due to anatomic anomalies such as diaphragmatic hernia, paraesophageal hernia, diaphragmatic eventration, or phrenic nerve paralysis. Among adults, paraesophageal hernia is the most common diaphragmatic defect associated with gastric volvulus [1].

Clinical presentation varies, with symptoms ranging from persistent nausea, vomiting, and chest pain to abdominal pain. Patients with a history of hiatal hernia who present with persistent vomiting despite antiemetic treatment should raise suspicion for gastric volvulus [2]. Given its rarity, gastric volvulus necessitates a high index of suspicion, as delayed diagnosis may lead to severe complications such as gastric infarction, perforation, septic shock, and potentially fatal outcomes [2]. Mortality rates associated with gastric necrosis and perforation can be as high as 60% [4,5]. By itself, gastric volvulus is associated with a mortality rate of 30 to 50% [6].

Diagnostic evaluation typically involves plain radiographs, oral contrast studies, or CT scans [6]. However, an upper gastrointestinal contrast study and an upper gastrointestinal endoscopy are the most useful tests. These methods have a diagnostic accuracy of 81% to 84% [7]. Management includes prompt fluid resuscitation, nasogastric (NG) tube placement, and endoscopy-guided NG tube placement in cases where traditional insertion methods fail. Surgical intervention, whether open or laparoscopic, is warranted in cases with signs of gastric mucosal ischemia, perforation, or sepsis. In secondary gastric volvulus, the diaphragmatic defect and gastropexy are repaired [6]. The upper gastrointestinal (GI) study findings for gastric volvulus may encompass a distended stomach in the left upper quadrant extending into the thorax, stomach inversion, luminal obstruction caused by a twist exceeding 180°, incomplete or absent contrast entrance/exit indicating acute obstructive volvulus, "beaking" observed at the twist point, and a mesenteroaxial orientation with the antrum and pylorus positioned above the gastric fundus.

## Case presentation

**Figure 1:**
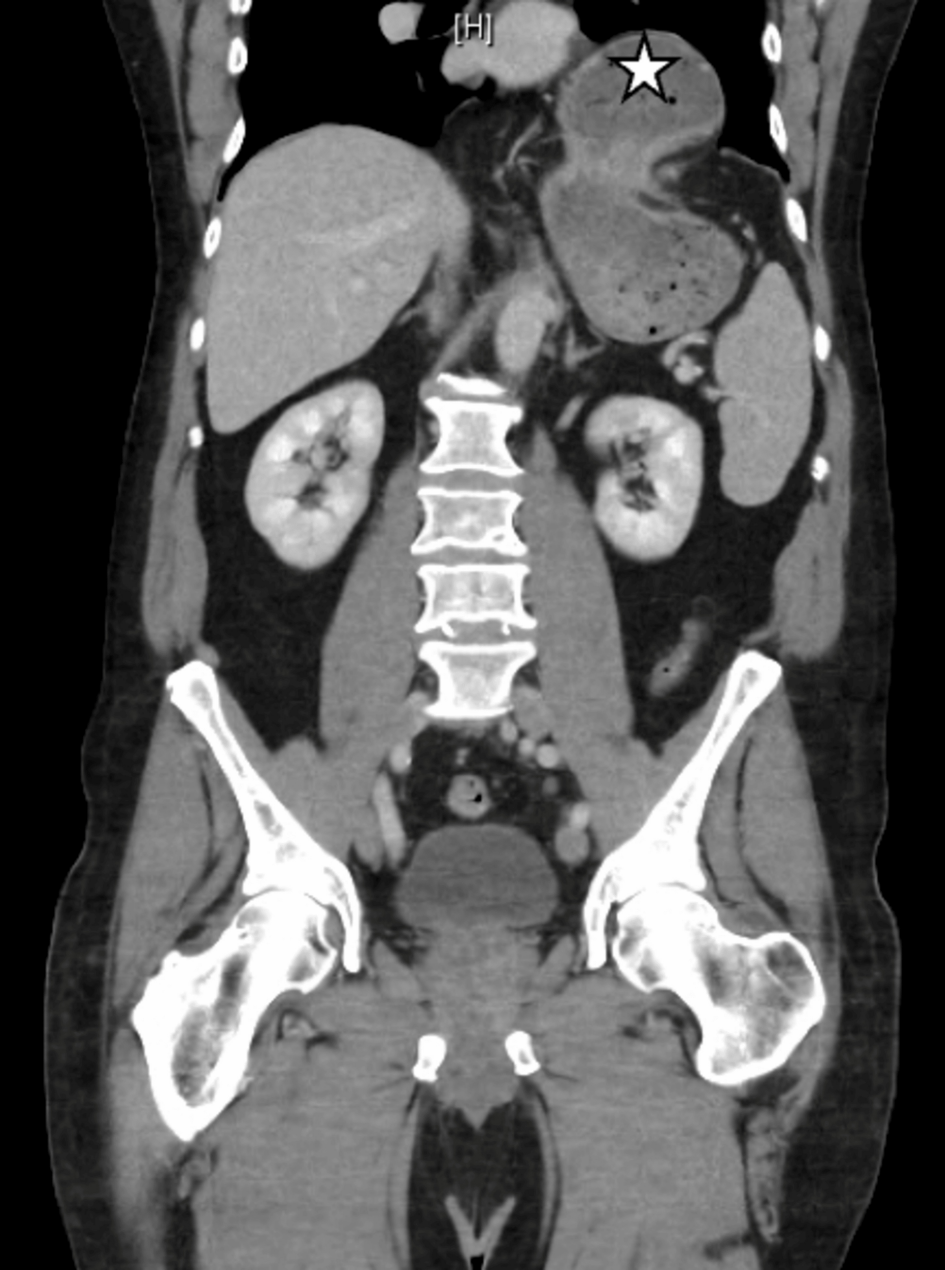
CT scan image of the abdomen The image demonstrates a large hiatal hernia (marked with a star).

A 71-year-old male with a medical history of umbilical hernia repair with mesh, hypertension, and gastroesophageal reflux disease (GERD) presented to the Emergency Room complaining of chest and abdominal pain accompanied by vomiting. The onset of symptoms occurred after dinner, progressively worsening with associated bloating and recurrent vomiting. No evidence of gastrointestinal bleeding was reported. An initial CT scan revealed protrusion of a significant portion of the stomach through the diaphragm into the chest cavity without signs of obstruction or perforation (Figure 1). The patient was admitted with a suspected diagnosis of gastroenteritis and dysphagia. 

**Figure 2:**
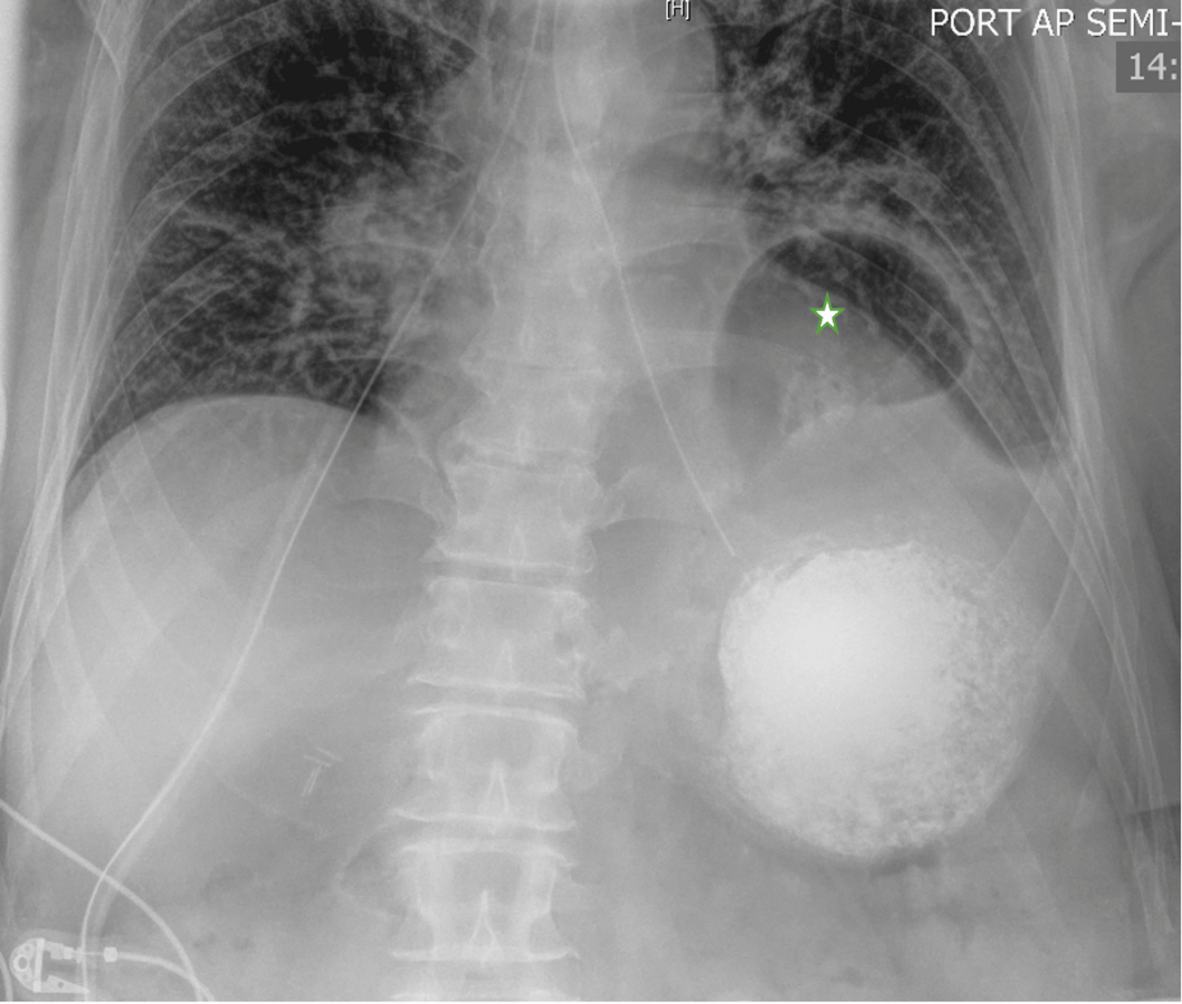
Abdominal X-ray image The image displays a hiatal hernia with a substantial portion of the stomach positioned above the left hemidiaphragm (marked with a star).

**Figure 3:**
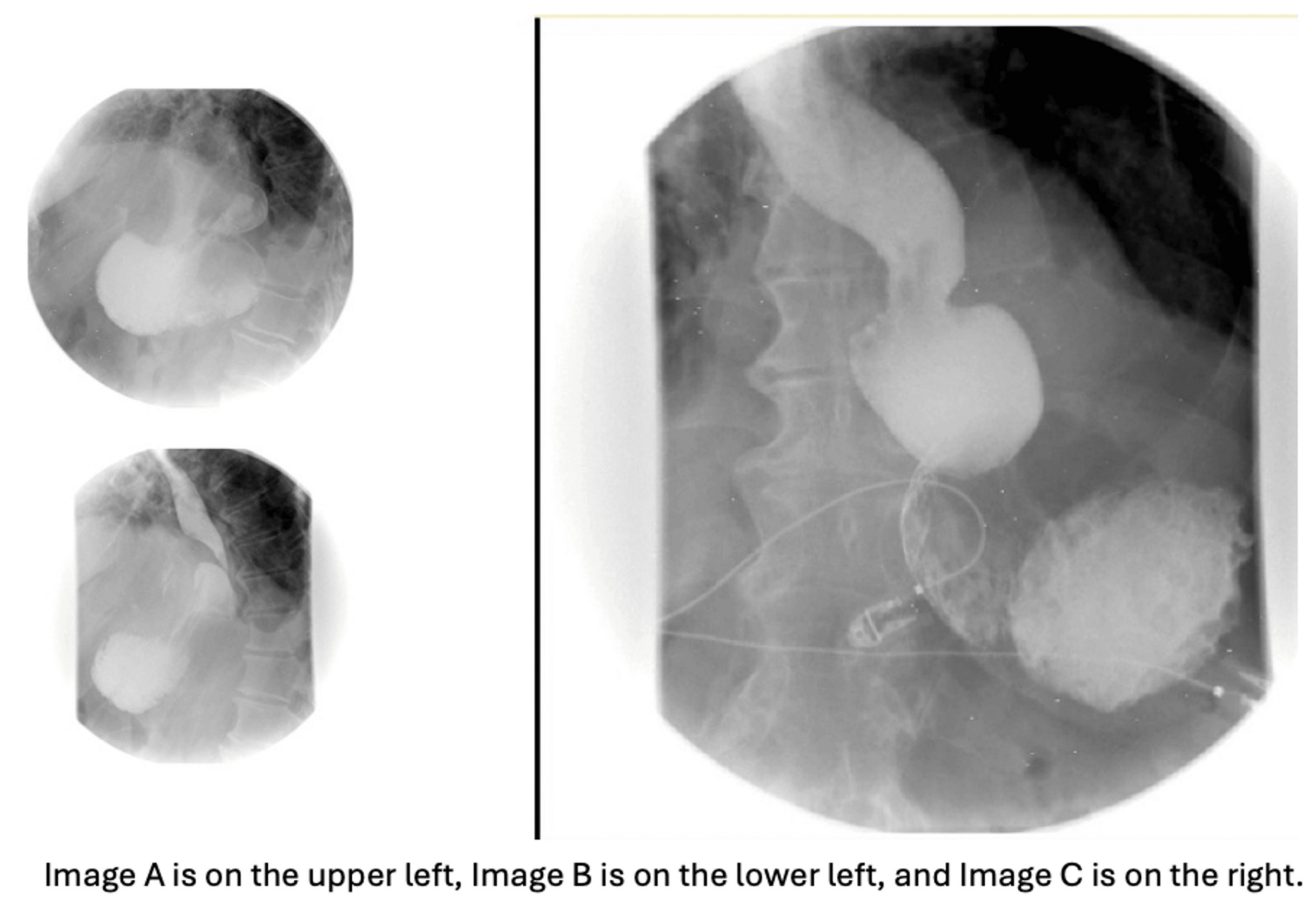
Barium esophagogram Images Sequential images from a barium esophagogram study. Images A and B reveal a hiatal hernia, while Image C shows no exit of contrast material from the stomach, indicating acute gastric volvulus.

Despite initial improvement on the first day of hospitalization, symptoms recurred after breakfast. The patient experienced persistent nausea and vomiting despite antiemetic treatment. A nasogastric tube was placed for stomach decompression. A follow-up X-ray of the abdomen revealed a prominent hiatal hernia with a large portion of the stomach above the left hemidiaphragm (Figure 2). The barium esophagogram showed a large sliding hiatal hernia, with the stomach containing fluid and debris despite the patient fasting for 10 hours, barium esophagogram also revealed no exit of contrast from the stomach indicating acute gastric volvulus (Figure 3). The patient was diagnosed with an incarcerated paraesophageal hernia and organoaxial gastric volvulus.

Urgent laparoscopic surgery was performed, confirming gastric volvulus with outlet obstruction due to hiatal hernia. The patient underwent laparoscopic paraesophageal hernia repair with Dor fundoplication and gastropexy, with no evidence of gastric ischemia. The patient did not have any postoperative complications.

## Discussion

Diagnosing gastric volvulus can pose a challenge due to its nonspecific symptoms, often leading to delayed identification. The Borchardt triad, characterized by pain, retching, and difficulty passing a nasogastric tube, aids diagnosis and is present in up to 70% of patients with gastric volvulus [2]. Additional indicators such as gas-filled viscus on radiographs and obstruction on upper GI series further support diagnosis. Imaging techniques such as radiography and computed tomography play a crucial role in confirming the diagnosis, complemented by findings from upper GI endoscopy.

Emergency surgical repair is the mainstay of treatment for acute gastric volvulus, although endoscopic reduction may be considered in specific cases. Prompt recognition and intervention are essential in averting complications associated with gastric volvulus. Patients with mild symptoms can be managed electively. However, those with complete obstruction need urgent care, as delays can increase the risk of gastric ischemia and mortality. Initial treatment includes intravenous fluid resuscitation and gastrointestinal endoscopy. Options for managing acute gastric volvulus include endoscopic or laparoscopic repair [5,8,9], a combined laparoendoscopic approach, and open procedures. To prevent the recurrence of gastric volvulus, gastropexy and paraesophageal hernia (PEH) repair are utilized. The Dor fundoplication and gastropexy are preferred surgical techniques in the management of gastric volvulus and associated hiatal hernias due to their effectiveness in preventing recurrence and addressing the underlying anatomical issues [10]. Laparoscopy is preferred even in urgent situations, providing superior visualization of the esophageal hiatus and posterior mediastinum compared to open surgery [10].

## Conclusions

We present a case of gastric volvulus where the patient initially exhibited mild signs of obstruction, such as nausea and vomiting. However, the paraesophageal hernia eventually led to acute gastric volvulus. Timely imaging studies were crucial for diagnosis. This case underscores the importance of maintaining a high suspicion for gastric volvulus in similar presentations. Diagnosing gastric volvulus requires suspicion and a barium study for confirmation. Clinicians should promptly order imaging when patients with hiatal hernia present with severe abdominal pain. Undiagnosed gastric volvulus poses a high risk of gastric ischemia, leading to serious complications and high mortality. Therefore, early diagnosis and immediate surgical intervention are essential for effective management.
